# Interactions between the discoidin domain receptor 1 and β1 integrin regulate attachment to collagen

**DOI:** 10.1242/bio.20135090

**Published:** 2013-09-13

**Authors:** Lisa A. Staudinger, Stephen J. Spano, Wilson Lee, Nuno Coelho, Dhaarmini Rajshankar, Michelle P. Bendeck, Tara Moriarty, Christopher A. McCulloch

**Affiliations:** 1 Matrix Dynamics Group, Faculty of Dentistry, University of Toronto, Toronto, ON M5S 3E2, Canada; 2 Department of Laboratory Medicine and Pathobiology, Faculty of Medicine, University of Toronto, Toronto, ON M5S 1A8, Canada

**Keywords:** Cell adhesions, Matrix remodeling, Phagocytosis

## Abstract

Collagen degradation by phagocytosis is essential for physiological collagen turnover and connective tissue homeostasis. The rate limiting step of phagocytosis is the binding of specific adhesion receptors, which include the integrins and discoidin domain receptors (DDR), to fibrillar collagen. While previous data suggest that these two receptors interact, the functional nature of these interactions is not defined. In mouse and human fibroblasts we examined the effects of DDR1 knockdown and over-expression on β1 integrin subunit function. DDR1 expression levels were positively associated with enhanced contraction of floating and attached collagen gels, increased collagen binding and increased collagen remodeling. In DDR1 over-expressing cells compared with control cells, there were increased numbers, area and length of focal adhesions immunostained for talin, paxillin, vinculin and activated β1 integrin. After treatment with the integrin-cleaving protease jararhagin, in comparison to controls, DDR1 over-expressing cells exhibited increased β1 integrin cleavage at the cell membrane, indicating that DDR1 over-expression affected the access and susceptibility of cell-surface β1 integrin to the protease. DDR1 over-expression was associated with increased glycosylation of the β1 integrin subunit, which when blocked by deoxymannojirimycin, reduced collagen binding. Collectively these data indicate that DDR1 regulates β1 integrin interactions with fibrillar collagen, which positively impacts the binding step of collagen phagocytosis and collagen remodeling.

## Introduction

Homeostasis of connective tissue in many organs is maintained through balanced synthesis and degradation of matrix proteins but is disrupted in fibrotic diseases. A critical process that contributes to connective tissue homeostasis is collagen degradation, which in physiological remodeling processes is mediated by phagocytosis of collagen fibrils (Everts et al., 1996). Collagen phagocytosis by fibroblasts is a receptor-driven process in which cellular recognition and binding to localized domains on collagen fibrils are crucial regulatory events in the phagocytic pathway (Chong et al., 2007; Knowles et al., 1991). Collagen recognition and attachment systems in fibroblasts include cell surface receptors with high affinity for collagen such as integrins (Knowles et al., 1991), specifically the α2β1 integrin. The α2β1 integrin is an important adhesion receptor for type I fibrillar collagen (Chong et al., 2007; Dickeson et al., 1999) and is also a critical determinant of the binding step of collagen phagocytosis (Arora et al., 2000; Lee et al., 1996).

The functional activity of β1 integrin receptors is affected by a broad range of regulatory molecules and processes including the concentration of divalent cations such as Ca^2+^ and Mg^2+^ (Schnapp, 2006), collagen structure and folding, and the clustering, allosteric modifications, post-translational modifications, organization and arrangement of integrins at cell membranes (Alberts, 2002). *N*-linked glycosylation is a post-translational regulatory mechanism for control of β1 integrin function (Bellis, 2004). Variations of β1 integrin glycosylation may influence receptor conformation (Bellis, 2004), surface expression (Akiyama et al., 1989; Hotchin and Watt, 1992), and receptor-mediated functional activity including cell adhesion and spreading on collagen (Diskin et al., 2009; von Lampe et al., 1993). Alterations in the oligosaccharide portion of integrins, which are mediated by glycosyltransferases such as GnT-III, GnT-V and α2,6 sialyltransferase, can regulate integrin-mediated cell migration and cell spreading (Gu and Taniguchi, 2008). Since β1 integrin ligand binding can be affected by variations of glycosylation (Gu et al., 2012), downstream signaling processes that regulate cell adhesion may also be affected, which includes the recruitment of actin binding proteins such as talin, paxillin and vinculin to focal adhesion complexes (Critchley, 2000; Keselowsky et al., 2004). While variations of normal glycosylation patterns of the β1 integrin have been identified in tumor cells (Bellis, 2004), the role of integrin glycosylation in regulating collagen binding and phagocytic function has not been described.

In addition to fibrillar collagen-binding integrins, discoidin domain receptors (DDRs) are a separate family of collagen-specific receptors that exhibit tyrosine kinase activity after ligand binding (Leitinger, 2011). DDR1 is activated by many types of collagens and appears to act as a sensor that triggers the degradation and turnover of extracellular matrix proteins (Franco et al., 2002; Leitinger, 2011). The biological importance of DDR1 in physiological matrix turnover is supported by experiments using genetic disruption that demonstrate a role for DDR1 in variety of fibrotic conditions of kidney (Flamant et al., 2006; Gross et al., 2010), liver (Song et al., 2011), lung (Avivi-Green et al., 2006) and blood vessels (Franco et al., 2010).

DDR1 is tyrosine phosphorylated and activated by cell binding to collagen, even in the presence of β1 integrin blocking antibodies, indicating that DDR1 can participate in signaling responses independent of β1 integrins (Vogel et al., 2000). Curiously, downstream signaling pathways activated by DDR1 can also intersect with β1 integrin-activated pathways (Valiathan et al., 2012). For example, activation of DDR1 inhibits integrin, FAK, and Cdc42-mediated cell spreading (Yeh et al., 2009) and integrin and STAT1/3-mediated cell migration (Yeh et al., 2011). After stimulation with type I collagen, β1 integrin activates Gli-1 whereas DDR1 activation stimulates the extracellular regulated kinase; combined activation of these two proteins enhances Bmi-1, which drives cell proliferation (Suh and Han, 2011). In pancreatic cancer cells, coordination of DDR1 and β1 integrin signaling can induce N-cadherin trafficking to the cell membrane, cell scattering and epithelial to mesenchymal transition in response to type I collagen (Shintani et al., 2008).

While DDRs can positively modulate integrin-mediated cell adhesion (Xu et al., 2012), there are no data to indicate that DDRs regulate integrin activation. It is not known whether the β1 integrin and DDR1 functionally interact to regulate adhesion to collagen and integrin function that controls the binding step of collagen phagocytosis. Accordingly we have examined the association and functional relationships between DDR1 and β1 integrin and the potential role of DDR1 in modulating collagen adhesions by regulating post-translational modifications of the β1 integrin.

## Materials and Methods

### Reagents

Rabbit monoclonal antibody to DDR1 (D1G6) was obtained from Cell Signaling Technology (Danvers, MA). Rabbit polyclonal antibody to DDR1 (C-20, sc-532) was obtained from Santa Cruz Biotechnology (Santa Cruz, CA). Rabbit polyclonal antibody to mouse β1 integrin (cytosolic domain), mouse monoclonal antibody to glyceraldehyde-3-phosphate dehydrogenase (GAPDH; clone 6C5) and mouse monoclonal antibody to paxillin (clone 5H11) were obtained from Millipore (Billerica, MA). Mouse monoclonal antibody to vinculin (clone hVIN-1), mouse monoclonal antibody to talin (clone 8d4), jararhagin snake venom from *Bothrops jararaca*, the selective inhibitor of α-mannosidase I, deoxymannojirimycin, dimethyl sulphoxide Hybri-Max^TM^, and protease inhibitor cocktail were obtained from Sigma-Aldrich (Oakville, ON). Rat monoclonal antibody to mouse CD29 (clone 9EG7), hamster monoclonal antibody to mouse CD29 (clone HM β1-1), rat monoclonal antibody to mouse CD29 (KMI6), and type I rat tail collagen were purchased from BD Biosciences (Mississauga, ON). Rabbit monoclonal antibody to vimentin (EPR3776) was purchased from Epitomics (Burlingame, CA). FITC-conjugated streptavidin and Cy3-conjugated streptavidin were from Cedarlane (Burlington, ON). Biotin-conjugated mouse monoclonal (2A 8F4) antibody to rat IgG2a-heavy chain was purchased from Abcam (Cambridge, MA). FITC-conjugated, mouse monoclonal antibody to rat IgG2a (MRG2a-83) and Alexa Fluor® 647-conjugated Armenian hamster IgG antibody to mouse CD49b (HMα2) were purchased from BioLegend (San Diego, CA). Goat antibody to mouse and goat antibody to rabbit IgG (H+L) conjugated to HRP were purchased from Bio-Rad (Hercules, CA). Fluoresbrite® microparticles (1 µm diameter yellow-green beads) were purchased from Polysciences (Warrington, PA) and crimson beads (1 µm diameter) were from InVitrogen (Burlington, ON). Endoglycosidase-H was purchased from Roche (Mannheim, Germany). Nilotinib was obtained from Reagents Direct (Encinitas, CA) and used at 1 µM in collagen bead binding experiments to inhibit DDR1 kinase activity as described (Manley et al., 2010).

### Cell culture

Mouse NIH-3T3 wild type cells, 3T3 cells transiently transfected with control vector and 3T3 cells stably transfected with DDR1 (b-isoform; obtained from the late Wolfgang Vogel; University of Toronto) were used. In some experiments, 3T3 cells or human gingival fibroblasts derived from adult gingival biopsies were treated with DDR1 knockdown (siRNAs for mouse and human DDR1 from ThermoScientific, Mississigauga, ON). In several experiments, β1 integrin-deficient GD25 cells (with and without siRNA knockdown of DDR1) were used (provided by Dr Reinhard Fässler; Max-Planck Institute for Biochemistry, Munich, Germany). Cells were cultured at 37°C in complete DME medium containing 10% fetal bovine serum and antibiotics. Cells were passaged with 0.05% trypsin with 0.53 mM EDTA.

### Immunoblotting

Whole cell extracts were prepared on ice by rinsing with cold Ca^2^- and Mg^2+^-free PBS before lysis with 1% TNT buffer (20 mM Tris pH 7.5, 150 mM NaCl, 1% Triton X-100, protease inhibitor cocktail, 200 mM NaVO_3_, 20 mg/mL PMSF). The homogenate was centrifuged at 14,000 g for 10 minutes at 4°C; the supernatant was retained for biochemical analysis of protein content by bicinchoninic acid analysis. Equal amounts of protein (10 µg per lane) were separated on 9% sodium dodecyl sulfate polyacrylamide gel electrophoresis gels, transferred to membranes and probed with appropriate antibodies. Immunoblots were quantified by scanning densitometry and ImageJ software.

### Flow cytometry

Cells were seeded on to collagen-coated (0.5, 1, 2 mg/mL) or uncoated non-tissue culture plastic for indicated lengths of time. To measure β1 integrin activation, cells were immunostained with the neo-epitope antibody, 9EG7 (Lenter et al., 1993), which recognizes activated β1 integrins. Cells were immunostained and analyzed by flow cytometry as described (Kim et al., 2010a; Kim et al., 2010b). We measured total β1 integrin surface expression in unfixed and non-permeabilized cells immunostained with KMI6 antibody and analyzed by flow cytometry. Cell surface α2 and α5 integrins in unfixed and non-permeabilized cells were measured by flow cytometry.

### Collagen bead binding

We measured matrix protein binding to cells using carboxylate-modified fluorescent polystyrene beads (1 µm diameter; yellow-green-excitation/emission- 441/486 nm (FITC beads); or crimson-excitation/emission- 625/645 nm crimson beads) that were coated with either type I fibrillar collagen (3.66 mg/mL; polymerization induced by incubation of beads at pH 7.4) or fibronectin (10 µg/mL), or with a non-specific binding control, BSA (0.66 mg/mL). Cells were seeded overnight on to tissue culture plastic. Collagen- or fibronectin-coated FITC beads were loaded on to the dorsal surface of cells together with BSA-coated crimson beads (6 of each bead type/cell, unless otherwise indicated) and incubated for 1 hour at 37°C, unless otherwise specified. Bead binding was analyzed by flow cytometry (10,000 cells per run) (Knowles et al., 1991).

In an experiment of similar design, cells were seeded overnight on to tissue culture-treated 8-well chamber slides (20,000 cells/well). Collagen-coated FITC beads were loaded on to the dorsal surface of cells together with BSA-coated crimson beads (60 of each bead type/cell) and incubated for 1 hour at 37°C in the presence or absence of β1 integrin-blocking antibody (CD29; 62.5 µg/mL) or pre-immune serum as a control. Following bead binding cells were rinsed with PBS, fixed with 4% paraformaldehyde and counter-stained with DAPI. By fluorescence microscopy, three separate fields of view were randomly selected and a minimum of 20 DAPI-stained nuclei and associated beads were counted. The mean±S.E.M. of the percentage of the cell population with bound beads was calculated.

### Collagen remodeling

Collagen was applied to plasma-treated, glass-bottom microwell dishes (35 mm dishes with 14 mm microwell inserts, MatTek) and polymerized at pH = 7.4 buffer. The collagen consisted of a 50:50 mixture of fibrillar rat tail collagen (1 mg/mL, BD Biosciences) and DQ type I collagen prepared from bovine skin (1 mg/mL, InVitrogen). Cells (10,000 per well) were incubated for 12 or 24 h at 37°C. After incubation the samples were rinsed twice with PBS, fixed in 3.7% formaldehyde, blocked with 1% BSA and stained for 30 min at 37°C with rhodamine phalloidin (Invitrogen). At least three separate fields for each sample were imaged by confocal microscopy (Leica) and the images were quantified to estimate the remodeling activity of the different cells with Image J. Remodeling was quantified from the fluorescence intensities of FITC-labeled collagen in fixed area, regions of interest in each sample. Fluorescence was normalized for samples of collagen gels without cells.

### Collagen gel contraction

Cells were added to polymerizing collagen solutions (1.36 mg/mL; 150,000 cells/mL). Aliquots (0.2 mL) were pipetted on to the center of each well of a 24 well non-tissue culture plate. A floating gel contraction protocol was used to measure collagen remodeling (Nakagawa et al., 1989). Measurements were made of the collagen gel diameters with a dissecting microscope and an interocular grid at 0 hrs and every 10–12 hrs for a total of 72 hours. We examined cell-mediated collagen contraction with an attached gel contraction assay (Nakagawa et al., 1989). Collagen gels containing cells were incubated at 37°C for 3 days prior to release from the base of the dish with a pipette. Measurements of gel diameter were made at 0 min and every 30 mins thereafter until contraction stopped. Gel diameters from three separate floating and attached gel contraction experiments were plotted over time. Linear regression was used to estimate the rate of collagen contraction.

### Cell migration

Cells in monolayers were seeded on to type I rat tail collagen-coated plates (1 mg/mL) (Chou et al., 1996). A scratch in the monolayer was created using a 200 µl pipette tip to study migration into the denuded space (Coomber and Gotlieb, 1990; Zahm et al., 1997). Following the scratch, phase-contrast images were obtained every 2 hours for 12 hours to measure cell migration back into the cell denuded area. The change in width of the scratch for three separate experiments was plotted over time and slopes fitted with linear regression.

### Immunostaining

Cells were plated overnight on type I fibrillar collagen-coated (1 mg/mL) (Chou et al., 1996) glass coverslips (MatTek dishes; Ashland, MA) to enable spreading and formation of focal adhesions. Cells were fixed with 4% paraformaldehyde in PBS for 10 min, permeabilized with 0.2% Triton X-100 in PBS, and blocked for 1 hr in 0.2% BSA in PBS. Cells were incubated with primary antibody and FITC-conjugated secondary antibody for 1 hr each. Immunostained cells were visualized by total internal reflection fluorescence (TIRF) microscopy (Leica, Heidelberg, Germany; 100× objective; numerical aperture = 1.45). Focal adhesion proteins in contact with the collagen substrate (optical penetration depth <110 nm) were analyzed. The mean number of focal adhesions in the cell periphery (<5 µm from the cell membrane) and in the cell body (>5 µm from the cell membrane) were quantified with MetaMorph® (20 cells/condition; Leica Microsystems, Wetzlar, Germany).

### Integrin cleavage

Cells in monolayers on non-tissue culture plastic coated with polymerized type I collagen were treated with 10 µg/mL of jararhagin in growth medium for 1 hr at 37°C (Klein et al., 2011). After 1 hr cells were immediately placed on ice and the jararhagin-containing medium was removed. Cells were prepared by rinsing with cold Ca^2+^- and Mg^2+^- free PBS before lysis in 1% TNT buffer (20 mM Tris pH 7.5, 150 mM NaCl, 1% Triton X-100, protease inhibitor cocktail, 200 mM NaVO_3_, 20 mg/mL PMSF). Protein concentrations were measured using a BCA assay prior to immunoblot analysis.

### Glycosylation

Cells in monolayers were grown to 90% confluence on uncoated tissue culture plastic or non-tissue culture plastic coated with 200 µg/mL of type I rat tail collagen neutralized to pH 7.5. Cells were rinsed with cold Ca^2+^-, Mg^2+^-free PBS prior to lysis with extraction buffer (1% Triton X-100, 0.125% Tween-20, 0.5% deoxycholate, 50 mM HEPES, 0.5 M NaCl pH 7.5, protease inhibitor cocktail, 200 mM NaVO_3_, 20 mg/mL PMSF). Protein concentrations were measured using a BCA assay and 10 µg/µL of glycoprotein sample were combined with 5× reaction buffer (250 mM sodium phosphate buffer), denaturation solution (0.02% SDS, 0.1 M β-ME), PMSF and protease inhibitors. Samples were heated at 100°C for 3 min and endoglycosidase-H was added to obtain a final enzyme concentration of 0.25 mU/µL followed by incubation at 37°C for 24 h. The reaction was terminated by ice-cold acetone/trichloroacetic acid precipitation. The pelleted samples were air dried prior to the addition of sample loading buffer and subsequent immunoblot analysis (Akiyama et al., 1989; Salicioni et al., 2004). In some experiments, a selective inhibitor of α–mannosidase I, deoxymannojirimycin (MNJ) (Esko and Bertozzi, 2009) was used to inhibit glycosylation in intact cells.

### Statistics

For all data, mean and standard errors of means were computed. The mean slopes of gel contraction assays were computed by best fit of linear regression for each experiment. When appropriate, comparisons between two samples were made by Student's t-test or for multiple samples with analysis of variance followed by Tukey's test for assessing individual differences. Statistical significance was set at a type I error rate of *P*<0.05. All experiments were performed in triplicate unless otherwise stated.

## Results

### Cell characterization

DDR1 was expressed at low levels in NIH 3T3 wild type cells and in 3T3 wild type cells transfected with empty vector (controls) but was expressed at >8-fold higher levels in DDR1 over-expressing cells compared with wild type cells (supplementary material Fig. S1A). DDR1 was not detectable in 3T3 cells with DDR1 knockdown. DDR1 was abundantly expressed in human gingival fibroblasts but was not detectable in cultures treated with siRNA (supplementary material Fig. S1B). β1 integrin was expressed in wild type and DDR1 over-expressing cells but not in GD25 cells (supplementary material Fig. S1C).

### Collagen binding to β1 integrins is enhanced by DDR1 over-expression

**Fig. 1.**
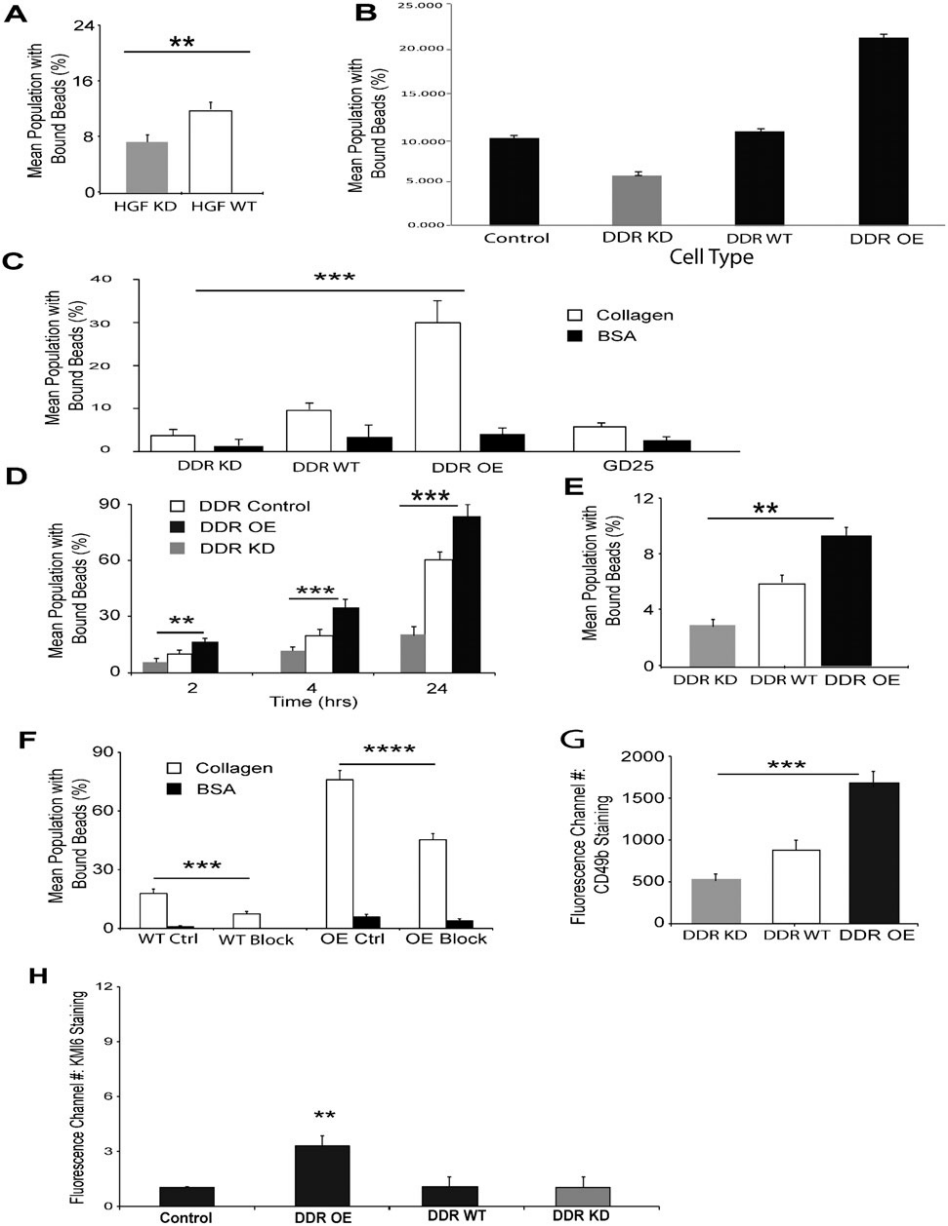
Collagen binding to β1 integrins is regulated by DDR1 expression levels. (A) Human gingival fibroblasts treated with siRNA for DDR1 (HGF KD) or with irrelevant siRNA (HGF WT) were incubated with fluorescent collagen-coated beads for 1 hr. Bead binding was analyzed by flow cytometry. Data are mean±standard errors of % of cells that bound collagen beads. (*n* = 3 independent experiments). ** indicates difference of *P*<0.01. (B) Binding of collagen-coated beads to indicated cell types was analyzed by flow cytometry. Cells were incubated with beads for 2 hrs. Data are mean±standard errors of % of cells that bound collagen beads. (*n* = 3 independent experiments). (C) Binding of collagen-coated beads to indicated cell types was analyzed by flow cytometry 2 hrs after incubation. Cell binding to BSA-coated crimson beads was measured simultaneously as a non-specific binding control. Data represent mean±S.E.M. (*n* = 3 independent experiments). *** indicates difference of *P*<0.001. (D) Flow cytometric analysis to determine collagen-coated bead binding to DDR1 knockdown and over-expressing cells. Data represent mean±S.E.M. (*n* = 3 independent experiments). ** and *** indicate differences of *P*<0.01 and *P*<0.001 between indicated groups. (E) Binding of fibronectin-coated beads to DDR1 knockdown, wild type and over-expressing cells was measured by flow cytometry. Data represent mean±S.E.M. (*n* = 3 independent experiments). ** indicates difference of *P*<0.01. (F) Cells were plated overnight in 8-well chamber slides. Binding of collagen-coated beads in the presence of pre-immune serum or CD29 blocking antibody (62.5 µg/mL) was analyzed after 1 hour incubation. Mean percentage of cell population with bound beads was determined by counting beads overlying cells and the number of DAPI-stained nuclei. Collagen coated-bead binding to cells was reduced by CD29 blocking antibody in empty vector control (*P*<0.001) and DDR1 over-expressing (*P*<0.0001) cells. BSA-coated crimson beads were measured simultaneously as non-specific binding controls. Three separate fields of view were randomly selected and a minimum of 20 DAPI-stained nuclei and associated beads were counted. Data represent mean±S.E.M. (*n* = 4 independent experiments). (G) Cells were plated overnight on tissue culture plastic. α2 integrin surface abundance was measured by immunostaining of non-fixed and non-permeabilized cells with antibody to CD49b, followed by flow cytometry. Data represent mean±S.E.M. of fluorescence channel number of cells stained with fluorescence-conjugated antibodies (*n* = 3 independent experiments). *** indicates difference of *P*<0.001. (H) Indicated cell types were plated on collagen-coated tissue culture plastic overnight, detached with versene, not permeabilized, immunostained with KMI6 for cell surface β1 integrin abundance and analyzed by flow cytometry. ** indicates difference of *P*<0.01.

We first determined whether DDR1 levels affect cell adhesion to collagen by bead binding and flow cytometry analysis. In two hour binding assays, collagen-coated bead binding to human gingival fibroblasts treated with siRNA for DDR1 was reduced by ∼40% compared to cells treated with irrelevant siRNA (Fig. 1A; *P*<0.01). Collagen bead binding in DDR1 over-expressing 3T3 cells was >2-fold higher (*P*<0.001) than DDR1 control cells or DDR1 wild type cells while cells with DDR1 knockdown exhibited ∼50% less bead binding than wild-type cells (Fig. 1B).

In similar types of bead binding assays, DDR1 over-expressing cells exhibited >3-fold higher collagen bead binding than wild-type cells and >5-fold higher (*P*<0.001) than GD25 cells that do not express β1 integrins (Fig. 1C). Binding of BSA-coated beads to the different cell types was <4% for all cell types. In a time-course experiment of similar design, we found that collagen-coated bead binding to DDR1 over-expressing cells was consistently higher than wild type cells for all time periods and DDR1 knockdown cells was consistently lower than the wild type cells (Fig. 1D). Collagen bead binding was not affected by the tyrosine kinase activity of DDR1 since inhibition of DDR1's kinase activity with nilotinib (1 µM; 1 hr incubation before bead binding; IC 50 = 3.7 nM) (Manley et al., 2010) did not affect the % of DDR1 over-expressing cells that bound collagen beads (*P*>0.2).

We determined whether over-expression of DDR1 affects the binding of a non-collagenous, β1 integrin-binding protein. As the α5β1 integrin is an important adhesion receptor for fibronectin (Zhang et al., 1993), we considered that expression levels of DDR1 may affect β1 integrin-dependent adhesion. Latex beads were coated with fibronectin (McKeown et al., 1990) and bead binding assays were conducted with DDR1 wild type, knockdown and over-expressing cells. Similar to the data for collagen bead binding, fibronectin-coated bead binding (1 hr) to DDR1 over-expressing cells was higher than wild type cells (1.6-fold; *P*<0.01) while binding was 50% lower in DDR1 knockdown cells compared with wild type (*P*<0.01; Fig. 1E).

We measured the relative contribution of collagen binding that was attributable to DDR1 over-expression versus enhancement of β1 integrin binding activity. Cells were plated overnight in 8-well chamber slides; binding of collagen-coated beads in the presence of pre-immune serum or β1 integrin blocking antibody was analyzed after 1 hour bead incubation. In wild type cells treated with blocking antibody, binding of collagen coated-beads to cells was reduced to 30% of levels in cells treated with pre-immune serum (*P*<0.001). In DDR1 over-expressing cells treated with blocking antibody, collagen bead binding was reduced to 60% of cells treated with pre-immune serum (*P*<0.0001; Fig. 1F). There were no significant changes of BSA-coated bead binding with these treatments (*P*>0.2).

Since α2β1 is the major integrin receptor for type I fibrillar collagen (Arora et al., 2000; Lee et al., 1996), we determined whether DDR1 enhances cell surface expression of the α2 integrin subunit. Immunostaining of non-permeabilized cells with antibody to the α2 integrin subunit (CD49b) and analysis by flow cytometry showed that expression of total cell surface α2 integrin was enhanced by 1.9-fold (*P*<0.0001) in DDR1 over-expressing cells compared with wild type cells and reduced by 35% in cells with DDR1 knockdown (Fig. 1G). Notably, the α11 integrin, which is another integrin sub-unit that binds fibrillar collagen (Barczyk et al., 2010), was not detectable by immunoblotting of wild type or DDR1 over-expressing cells (data not shown). We measured cell surface β1 integrin abundance in non-permeabilized DDR1 control, DDR1 over-expressing cells, DDR1 wild type and DDR1 knock down cells by immunostaining and flow cytometry. The fluorescence intensity attributable to β1 integrin staining was increased >2-fold in the DDR1 over-expressing cells compared with the other cell types (*P*<0.01; Fig. 1H). We also assessed cell surface α5 integrin sub-unit by flow cytometry analysis of non-permeabilized cells and found increased fluorescence intensity for the DDR1 over-expressing cells (DDR1 OE - 2.2±0.3 fluorescence channel number; DDR1 wild type - 0.6±0.1 fluorescence channel number; *P*<0.05).

### DDR1 over-expression enhances collagen remodeling and contraction

**Fig. 2.**
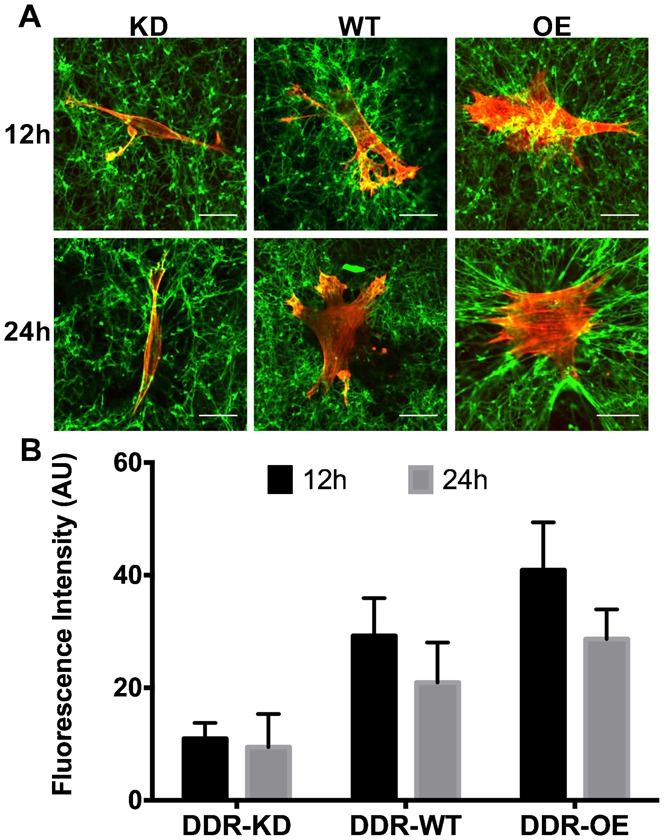
DDR1 over-expression enhances collagen fibril reorganization. (A) Knockdown (KD), wild type (WT) or DDR1 over-expressing (OE) cells were cultured on FITC-labeled fibrillar collagen gels for 12 h (upper panel) or 24 h (lower panel) to evaluate morphologically, collagen remodeling. Bar = 20 µm; (B) Quantification of FITC-collagen fluorescence in fixed area regions of interest by image analysis using Image J. Mean fluorescent intensity was obtained from average fluorescence intensities normalized to data of same gels without cells. At least 4 representative images were analyzed for each condition. Data are mean±standard errors of fluorescence intensity in arbitrary units (AU) in sampling grids.

Wild type, knockdown or DDR1 over-expressing cells were cultured on fluorescence-labeled type I fibrillar collagen gels for 12 h or 24 h. Microscopic evaluation showed that wild type and DDR1 over-expressing cells exhibited increased reorganization and compaction of collagen fibers at cell peripheries compared with DDR1 knockdown cells (Fig. 2A). Quantification of fluorescent intensity measurements of labeled collagen showed higher values of mean fluorescent intensities in wild type and DDR1 over-expressing cells compared with DDR1 knockdown cells (Fig. 2B), consistent with the fluorescent images of DDR1-enhanced collagen compaction and remodeling. Notably the DDR1 over-expressing cells exhibited marked reduction of collagen fluorescence (*P*<0.05) between 12 and 24 hrs, a reflection of increased collagenolysis by cells. In DDR1 knockdown cells, there was no change in collagen fluorescence between 12 and 24 hrs, reflecting the importance of DDR1 in mediating collagen compaction around cells.

A floating gel contraction assay was used to examine the ability of cells to reorganize collagen fibrils as a result of migratory activities (Grinnell, 2003) over 3 days. DDR1 over-expression enhanced floating collagen gel contraction compared with control and wild type (*P*<0.01) and by 15-fold (*P*<0.01) compared with GD25 cells and GD25 cells with DDR1 knockdown (Table 1). Knockdown of DDR1 expression was associated with reduced collagen gel contraction compared to wild type and control cells (*P*<0.05). Collagen gel contraction attributable to cell-mediated traction was measured with an attached gel contraction assay (Grinnell, 2003). DDR1 over-expression enhanced the contraction of attached collagen gels by >3-fold (*P*<0.01) compared with control and wild type and DDR1 knock down cells and by 4-fold (*P*<0.01) compared with GD25 cells and GD25 cells with DDR1 knockdown (Table 1). In cells with DDR1 knockdown, there was ∼35% reduction of gel contraction compared with wild type or control cells (*P*<0.05).

**Table 1.**
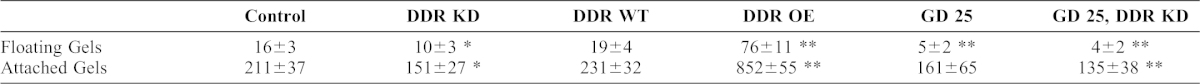
Cell-mediated contraction of floating and attached collagen gels. Cells were incubated in collagen solutions (10^6^ cells per ml of collagen) and the collagen was allowed to polymerize. For floating gels, the collagen gels were floated on cell culture medium. For the attached gels, collagen was allowed to adhere to plastic dishes; cells then developed tension in the gels prior to their release from the underlying dishes. The reduction of collagen gel diameter over time (fitted by linear regression) was used as a measure of cell-mediated contraction. Data are mean±standard error of the mean slope of the reduction of gel diameters over time (µm/hr). Significant differences between indicated groups and controls are marked by ** for *P*<0.01 and * for *P*<0.05.

### DDR1 expression and cell migration on collagen

Wild type or DDR1 over-expressing cells were grown to confluence on collagen-coated plastic prior to assays. Representative images obtained at 0 hours or 12 hours after scratching showed cell migration into the scratch “wound” and a reduction of the width of the denuded cell monolayer (supplementary material Fig. S2A). Cells over-expressing DDR1 showed slower migration than wild type cells (supplementary material Fig. S2B,C; *P*<0.05).

### DDR1 over-expression enhances activated β1 integrins in focal adhesions

**Fig. 3.**
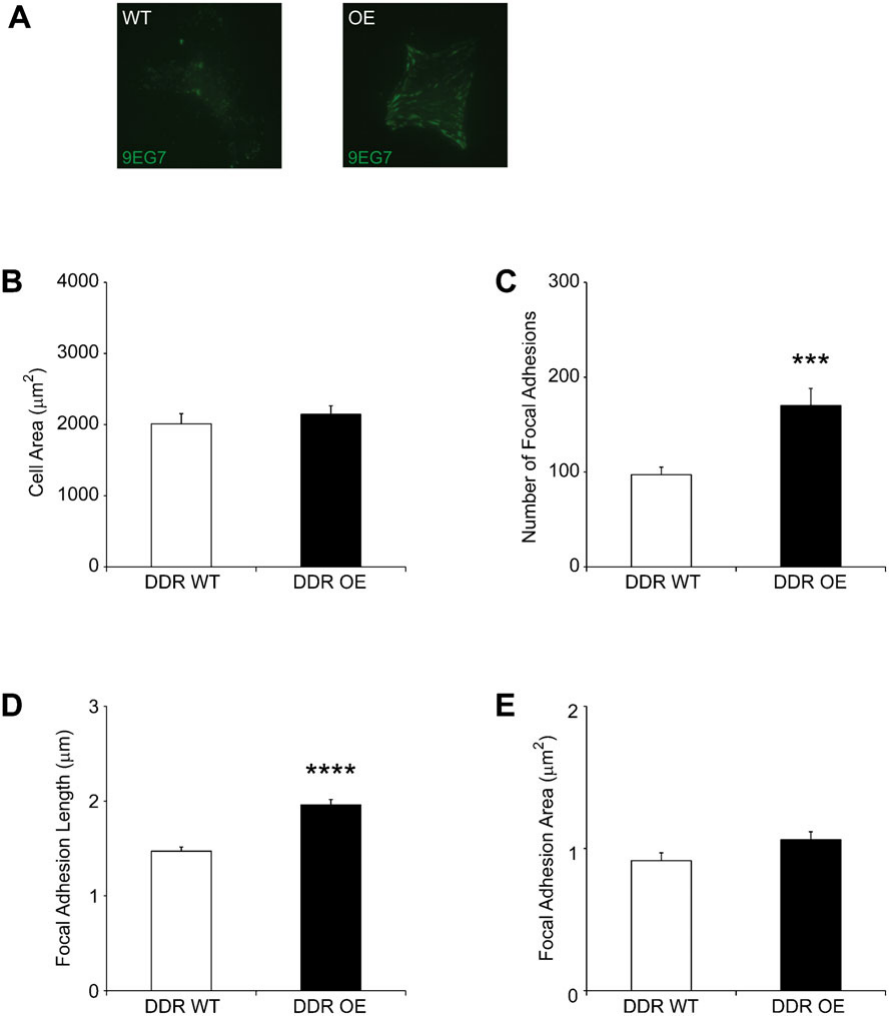
DDR1 over-expression enhances activated β1 integrin in focal adhesions. (A) Cells plated overnight on collagen were immunostained for activated β1 integrins using 9EG7 antibody and imaged by TIRF microscopy. Representative images indicate that DDR1 over-expressing cells expressed more activated β1 integrins than DDR1 wild type cells. (B) Metamorph was used to quantify mean±S.E.M. of cell area and focal adhesions in cells (*n* = 20 cells analyzed for each cell type). Total cell area estimated from peripheral 9EG7 staining in DDR1 over-expressing cells and wild type cells showed no significant difference in total cell area (*P* = 0.4807); (C) Increased numbers of activated β1 integrin-containing focal adhesions in DDR1 over-expressing cells (*P*<0.001); (D) Longer focal adhesions (*P*<0.0001) in DDR1 over-expressing cells; (E) No significant difference in focal adhesion area (*P* = 0.064).

Although DDR1 does not localize to focal adhesions (Vogel et al., 2000), we determined whether over-expression of DDR1 might influence the activation of β1 integrins and their recruitment to adhesions. Cells were plated overnight on fibrillar collagen, stained with 9EG7 (a neo-epitope antibody that binds to activated β1 integrins) (Lenter et al., 1993) and analyzed by TIRF microscopy (Rajshankar et al., 2012). Compared with wild type, DDR1 over-expressing cells contained more adhesions with activated β1 integrins (Fig. 3A; *P*<0.01). Image analysis that used thresholding of cell peripheries and computation of cell areas indicated that there was no significant difference of cell area between wild type and DDR1 over-expressing cells (Fig. 3B; *P* = 0.48). Analysis of focal adhesion staining in DDR1 over-expressing cells compared with wild type cells showed that the number and length of 9EG7-stained focal adhesions were increased 1.8 and 1.5-fold respectively (Fig. 3C,D; both by *P*<0.0001). The mean area of individual focal adhesions stained by 9EG7 was not significantly different (Fig. 3E; *P* = 0.064).

### DDR1 over-expression enhances focal adhesion maturation

**Fig. 4.**
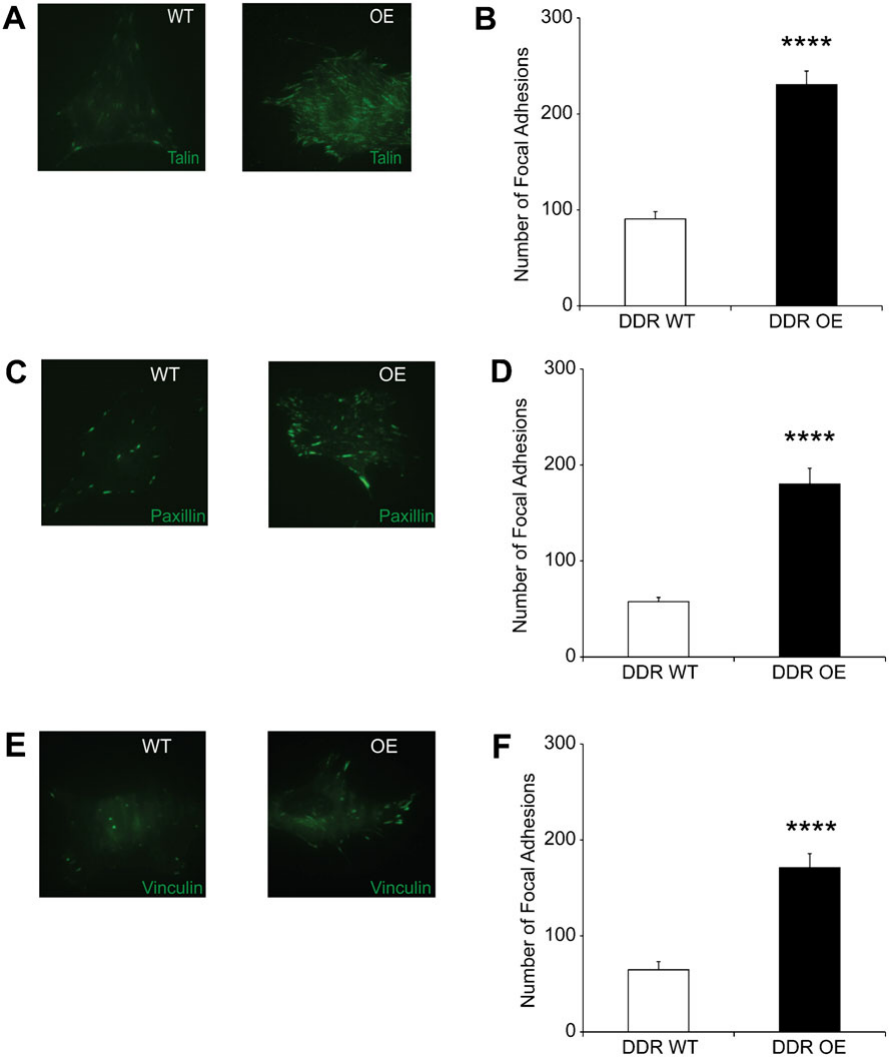
DDR1 over-expression enhances focal adhesion maturation. (A) Cells plated overnight on collagen were immunostained for talin and imaged by TIRF microscopy. (B) Analysis with Metamorph shows increased numbers of talin-containing focal adhesions in DDR1 over-expressing compared to wild type cells (*P*<0.0001). (C) Cells plated overnight on collagen were immunostained for paxillin and imaged by TIRF microscopy. (D) DDR1 over-expressing cells exhibit increased numbers of paxillin-containing focal adhesions (*P*<0.0001). (E) Cells plated overnight on collagen were immunostained for vinculin and imaged by TIRF microscopy. (F) Increased numbers of vinculin-stained focal adhesions in DDR1 over-expressing cells (*P*<0.0001).

Talin is an adaptor protein recruited to adhesions after the binding of β1 integrins to collagen (Craig and Johnson, 1996) and other matrix ligands. Consistent with the results of activated β1 integrin staining, representative images indicated that DDR1 over-expressing cells exhibited more talin-stained focal adhesions compared with wild type cells (Fig. 4A). Analysis of talin staining showed that DDR1 over-expressing cells exhibited >2.5-fold (Fig. 4B; *P*<0.0001) more focal adhesions, which were longer and of increased area compared with wild type cells (supplementary material Fig. S3A,B).

Paxillin is recruited to focal adhesions after talin (Jiang et al., 2003). Immunostaining and TIRF microscopy indicated that DDR1 over-expressing cells demonstrated 3-fold more paxillin-containing focal adhesions than wild type cells (Fig. 4C,D; *P*<0.001). Paxillin-stained adhesions were longer and larger in area than wild type cells (supplementary material Fig. S3C,D).

Vinculin is an adaptor protein that links β1 integrins to the actin cytoskeleton and is recruited to focal adhesions after the recruitment of talin and paxillin (Craig and Johnson, 1996; Critchley, 2000). DDR1 over-expressing cells exhibited >2.5-fold more vinculin containing focal adhesions than wild type cells (Fig. 4E,F; *P*<0.0001). Vinculin-stained focal adhesions in DDR1 over-expressing cells were longer and larger in area compared with wild type cells (supplementary material Fig. S3E,F).

### Impact of DDR1 over-expression on β1 integrin surface expression and activation

**Fig. 5.**
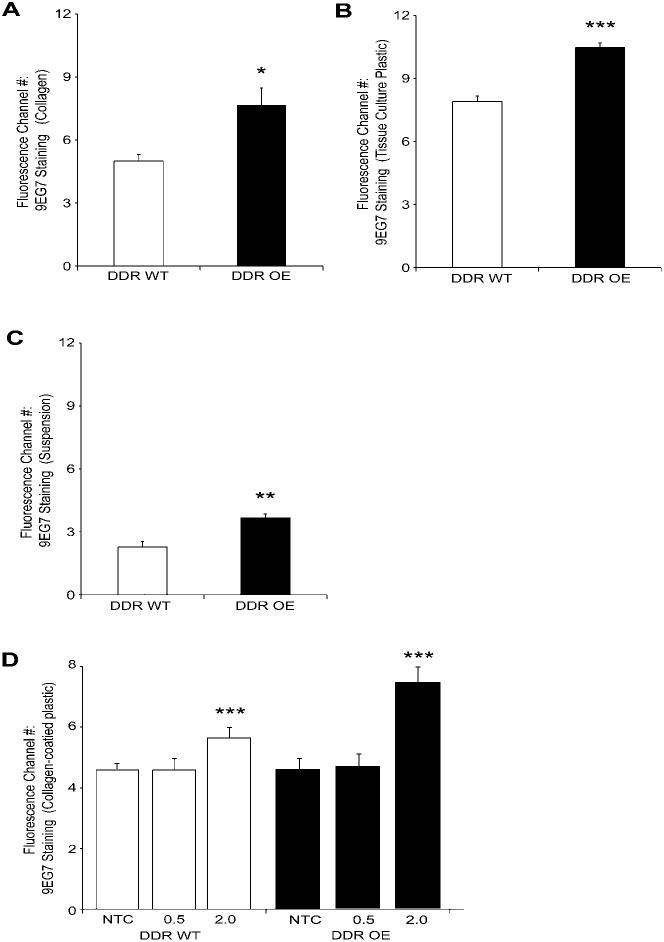
DDR1 over-expression enhances total β1 integrin surface expression and activation. (A) Cell surface activated β1 integrin on cells plated overnight on collagen. Immunostaining with 9EG7 antibody and analysis by flow cytometry of non-fixed, non-permeabilized cells. DDR1 over-expression increased total activated β1 integrin surface expression compared with wild type cells (*P*<0.05). (B) In cells plated on tissue culture plastic, staining of activated β1 integrin on cell surface (9EG7) is greater in DDR1 over-expressing cells than wild type cells (*P*<0.001). (C) Analysis of 9EG7 staining of cells in suspension. Activated β1 integrin on cell surface is greater in DDR1 over-expressing cells than wild type cells (**- *P*<0.01). Data are mean±S.E.M. (*n* = 3 independent experiments). (D) Same experimental approach as in panel C but cells were plated on non-tissue culture plastic, or non-tissue culture plastic coated with either 0.5 or 2 mg/ml collagen. (**- *P*<0.01). Data are mean±S.E.M. (*n* = 3 independent experiments).

By flow cytometry and immunostaining we measured activated β1 integrin on the whole cell surface with 9EG7 antibody. In cells plated overnight on fibrillar collagen, DDR1 over-expression increased 9EG7 binding by >1.5-fold (*P*<0.05) compared with wild type cells (Fig. 5A). Similar analysis of cells plated overnight on tissue culture plastic showed that 9EG7 staining was >1.3-fold (*P*<0.001) greater in DDR1 over-expressing cells than wild type cells (Fig. 5B). We deprived cells of attachment to matrix ligands by maintenance in suspension for 2 hours. Analysis of 9EG7 staining showed that total activated β1 integrin surface expression was >1.6-fold (*P*<0.01) greater in DDR1 over-expressing cells than wild type cells (Fig. 5C).

We determined whether increased concentrations of collagen on the substrate would affect the impact of DDR1 on β1 integrin activation in early phases of cell attachment. Wild type or DDR1 over-expressing cells were plated for 1 hr on non-tissue culture plastic, or on non-tissue culture plastic coated with 0.5 or 2 mg/ml collagen. Cells were quickly (<2 mins) detached with versene, fixed but not permeabilized, and immunostained with 9EG7 to estimate the relative abundance of activated β1 integrins on the cell surface. In cells plated on the higher concentration of collagen compared to non-tissue culture plastic or the lower concentration of collagen, there was increased β1 integrin activation in both cell types (*P*<0.01; Fig. 5D) but the activation was particularly enhanced in the DDR1 over-expressing cells.

### Effect of DDR1 on post-translational modifications of β1 integrin

**Fig. 6.**
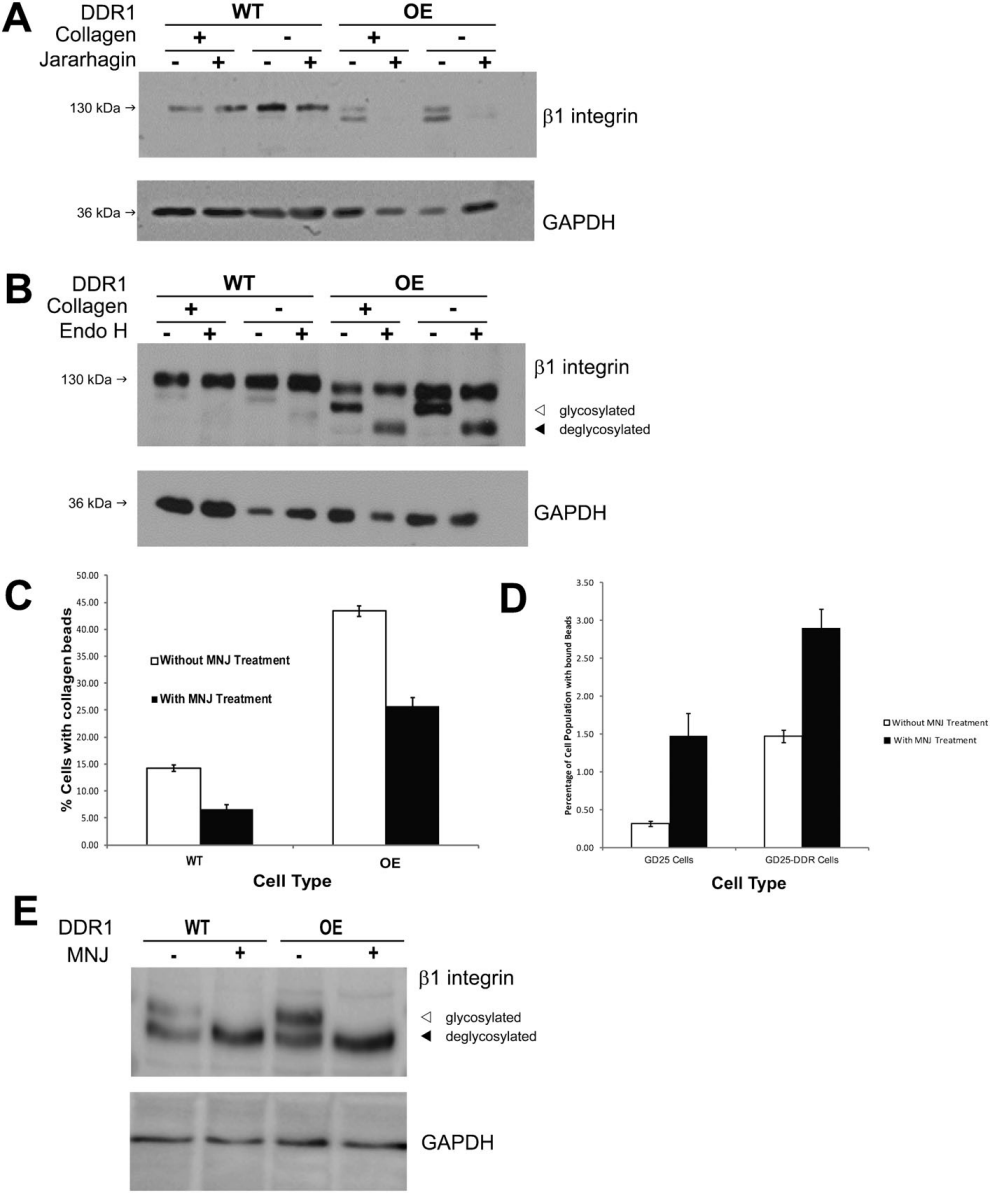
(A) DDR1 over-expression enhances enzymatic cleavage of β1 integrin at cell surface. Control or DDR1 over-expressing cells were plated on collagen (+) or tissue culture plastic (−). After 1 hr treatment with vehicle (−) or jararhagin (+) to induce cleavage of surface β1 integrins, whole cell lysates were prepared and immunoblotted for β1 integrin and GAPDH. Independent of the substrate used for cell plating, Immunoblot is representative of 3 independent experiments. (B) DDR1 over-expression affects glycosylation of β1 integrins. Control or DDR1 over-expressing cells were plated on collagen (+) or tissue culture plastic (−). Whole cell lysates were treated with vehicle (−) or endoglycosidase H (+) for 24 hrs and then immunoblotted for β1 integrin or GAPDH. Independent of the substrate used for cell plating, DDR1 over-expression was associated with increased abundance of glycosylated β1 integrin subunit, and by the band shift of the lower molecular weight band (denoted by arrows: ◃ glycosylated, ◂ deglycosylated) after treatment with endoglycosidase H. Representative immunoblot from 3 independent experiments. (C) Inhibition of α-mannosidase I reduces collagen bead binding. Indicated cell types were treated with deoxymannojirimycin (500 µg/ml, overnight) and then incubated with collagen beads for one hour. Collagen bead binding was assessed by flow cytometry as described above. Data are mean±S.E.M. (*n* = 3 independent experiments) of % of cells with bound collagen beads. (D) GD25 cells with and without knockdown of DDR1 were evaluated in the same experimental design as panel C. (E) Immunoblots of DDR1 wild type and DDR1 over-expressing cells that had been treated with deoxymannojirimycin (500 µg/ml, overnight). The lower molecular mass band represents the deglycosylated β1 integrin.

Cells were plated on tissue culture plastic or collagen and treated with the disintegrin jararhagin to induce cleavage of β1 integrin at the cell surface (Klein et al., 2011). After jararhagin treatment, whole cell lysates of wild type and DDR1 over-expressing cells were immunoblotted for β1 integrin (Fig. 6A). Independent of the substrate, cells that over-expressed DDR1 exhibited increased cleavage of the β1 integrin subunit after jararhagin treatment.

In immunoblots developed with longer exposures to visualize lower abundance proteins, we found a difference of the relative abundance of two different molecular mass bands of the β1 integrin in wild type cells compared with DDR1-over-expressing cells (supplementary material Fig. S1B). We considered that these bands may represent differences of post-translational modifications of the β1 integrin subunit (Bellis, 2004), including variations of glycosylation (Sun et al., 2009). We examined the relative abundance of β1 integrin subunit glycosylation in cells plated on collagen or tissue culture plastic. Cell lysates were treated with endoglycosidase-H and immunoblotted for the β1 integrin (Fig. 6B). Independent of the substrate on which cells were plated, cells that over-expressed DDR1 exhibited increased glycosylation of the β1 integrin subunit, which was suggested by a shift of the lower molecular mass band (∼125 kDa) to a band of ∼110 kDa after treatment with endoglycosidase-H.

Previous data have shown that the β1 integrin is extensively glycosylated as a result of processing in the Golgi and subsequent presentation on the cell surface (Gu and Isaji, 2008; Gu and Taniguchi, 2004; Gu and Taniguchi, 2008) and that glycosylation affects β1 integrin-dependent cell adhesion (Gu et al., 2012). We treated wild type and DDR1 over-expressing cells with deoxymannojirimycin, a selective inhibitor of α–mannosidase I (Esko and Bertozzi, 2009) to inhibit glycosylation in intact cells. For both cell types, collagen bead binding was reduced by ∼50% (Fig. 6C; *P*<0.001 for both cell types). As inhibition of α–mannosidase I could impact many different aspects of cell function, we performed similar collagen bead binding experiments using GD25 cells (with no β1 integrin expression) that expressed or were treated with siRNA to knock down DDR1. For both cell types, collagen bead binding was very low and, in contrast to cells that expressed β1 integrin, deoxymannojirimycin enhanced collagen bead binding (Fig. 6D), indicating that the inhibitory effect of deoxymannojirimycin on cell adhesion is dependent on the expression of the β1 integrin. Accordingly, we examined the effect of deoxymannojirimycin on the β1 integrin in wild type and DDR1 over-expressing cells by flow cytometry of β1 integrin abundance and by immunoblotting of cell lysates. While cells treated with deoxymannojirimycin exhibited a small increase of β1 integrin abundance at the surface of DDR1 over-expressing cells (Table 2), there was marked loss of the higher molecular mass (glycosylated) β1 integrin band in wild type and DDR1 over-expressing cells (Fig. 6E).

**Table 2.**
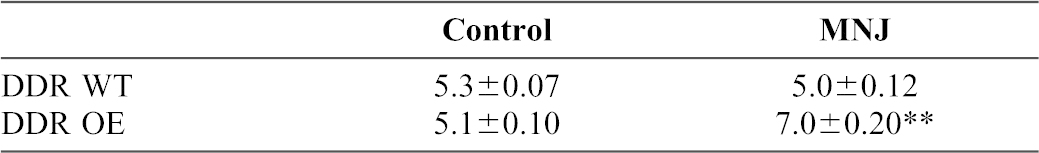
Cell surface abundance of β1 integrin measured by flow cytometry. DDR wild type and DDR over-expressing cells were treated with deoxymannojirimycin (MNJ) as indicated in the methods section. Cells were separated from plates with Versene and non-permeabilized, single cell suspensions were stained with KIM6 antibody that recognizes β1 integrin and counter-stained with FITC-conjugated goat anti-mouse antibody. Cells (10,000) were analyzed by flow cytometry and the mean±standard error of the mean fluorescence intensity are indicated. Significant differences of fluorescence intensities of β1 integrin staining between indicated groups are marked by ** for *P*<0.01.

## Discussion

Our principal finding is that DDR1 regulates β1 integrin interactions with fibrillar collagen, which extends recent observations on possible interactions between DDRs and the β1 integrin (Xu et al., 2012) and offers novel insights into the role of DDR1 in modifying β1 integrin function and post-translational modifications of the β1 integrin at the cell surface. In contrast to the recent study by Xu and co-workers, we found that DDR1 over-expression was associated with enhanced abundance and activation of cell surface β1 integrin expression, which we attribute to DDR1-mediated regulation of trafficking and presentation of cell surface β1 integrin. Further, in human gingival fibroblasts and 3T3 cells treated with DDR1 knockdown, we found that β1 integrin function was reduced. Taken together these data indicate that DDR1 expression levels regulate cell adhesion to collagen by regulating β1 integrin function.

### Collagen binding to β1 integrins is enhanced by DDR1 over-expression

As the α5β1 integrin is an important adhesion receptor for fibronectin (Hynes, 2002; Zhang et al., 1993), we determined whether DDR1 (which does not bind fibronectin) would also enhance fibronectin-coated bead binding. Our finding that DDR1 expression levels (over-expression and knockdown) regulated both type I collagen and fibronectin binding indicates that DDR1 regulates β1 integrin-dependent adhesion processes and that this regulation is independent of DDR1-mediated collagen adhesion.

We explored the role of DDR1 in regulating β1 integrin-mediated remodeling of collagen gels, an assay that provides insight into connective tissue remodeling by tractional and reorganizational processes (Grinnell, 2003). Our data from floating and attached collagen gel assays showed that DDR1 expression levels positively regulated β1 integrin functional interactions with fibrillar collagen, which is in contrast to earlier reports suggesting that DDR1 inhibits β1 integrin function (Yeh et al., 2009). The difference between our current data and the earlier findings of Yeh and co-workers may reflect the relatively higher levels of DDR1 expression in the cells that we used in our studies.

Earlier data showed that DDR1 does not localize to focal adhesions (Vogel et al., 2000). However, since we found that collagen binding was enhanced by DDR1 over-expression we determined whether the activation and recruitment of β1 integrins, and the enrichment of focal adhesions with the actin binding proteins talin, paxillin and vinculin, would be influenced. Our analyses of focal adhesion staining showed that after β1 integrin activation and ligand binding (Liu et al., 2000), talin (Craig and Johnson, 1996), paxillin (Jiang et al., 2003) and vinculin (Critchley, 2000) were recruited to focal adhesions. Further, in experiments using fluorescent collagen, we found that DDR1 expression levels (over-expression and knockdown) were strongly associated with collagen remodeling, collagen compaction and possibly later, collagenolysis. Collectively, these data underline our finding that DDR1 promotes binding and subsequent functional interactions of cells with collagen.

### DDR1 regulation of trafficking and post-translational modification of β1 integrin

We determined whether DDR1 affects β1 integrin trafficking to the cell surface by exploiting the susceptibility of cell surface β1 integrin to cleavage by the disintegrin jararhagin (Klein et al., 2011). Independent of whether cells were plated on collagen or tissue culture plastic, DDR1 over-expression was associated with increased cleavage of the β1 integrin subunit after treatment with jararhagin. The relative resistance of β1 integrins in wild type cells to both jararhagin and endoglycosidase H cleavage compared to over-expressing cells suggests that β1 integrin subunits in DDR1 over-expressing cells have a different conformation or presentation on the cell surface that enhances their susceptibility to cleavage by these enzymes.

In immunoblots with longer exposures we found differences of the relative abundance of different molecular mass β1 integrin subunits in wild type cells compared with DDR1-over-expressing cells. We considered that this variation may be due to differences in post-translational modifications of the β1 integrin subunit (Bellis, 2004; Diskin et al., 2009; Gu and Taniguchi, 2004), including variations of glycosylation. Independent of the substrate on which cells were plated, cells that over-expressed DDR1 exhibited increased *N*-linked glycosylation of the β1 integrin subunit, as indicated by the greater susceptibility of the faster migrating β1 band to endoglycosidase H. The functional significance of β1 integrin subunit glycosylation was studied in cells treated with deoxymannojirimycin, a selective inhibitor of α-mannosidase I. Both wild type and DDR1 over-expressing cells showed large reductions of collagen bead binding after treatment with deoxymannojirimycin. Further, the appearance of a single, lower molecular mass β1 integrin subunit band in immunoblots of these cell preparations indicated that glycosylation of the β1 integrin subunit is important in regulating cell adhesion. These data are consistent with earlier findings demonstrating that glycosylation is an important regulatory mechanism for β1 integrin function (Bellis, 2004; Gu et al., 2012; Gu and Taniguchi, 2008). Our findings of enhanced β1 integrin subunit glycosylation and abundance on the cell surface of cells over-expressing DDR1 are consistent with earlier data in which variations of β1 integrin glycosylation regulate integrin trafficking to the cell surface (Hotchin and Watt, 1992) and integrin-mediated cell migration (Janik et al., 2010), adhesion (von Lampe et al., 1993; Zheng et al., 1994), and spreading (Diskin et al., 2009).

In summary, our findings indicate that DDR1 expression levels regulate fibrillar collagen binding to β1 integrins in human and mouse fibroblasts. In cells over-expressing DDR1, β1 integrin cell surface expression and activation were dependent in part on DDR1-regulated post-translational modifications of the β1 integrin. These modifications in turn may translate into enhanced collagen phagocytosis and reorganization, processes that are essential for connective tissue homeostasis.

## Funding

Research was funded by a CIHR operating grant to C.A.M. [MOP418228], whose salary is supported by a Canada Research Chair (Tier 1).

## Supplementary Material

Supplementary Material
