# Supplementary material for: Interactions between the discoidin domain receptor 1 and β1 integrin regulate attachment to collagen
Source: Biol Open. 2013 Sep 13;2(11):1148–59. doi: 10.1242/bio.20135090 (PMC3828761; doi:10.1242/bio.20135090)
Supplement: Supplementary Material [file supp_2_11_1148__index.html]

Interactions between the discoidin domain receptor 1 and β1 integrin regulate attachment to collagen — Interactions between the discoidin domain receptor 1 and β1 integrin regulate attachment to collagen — Supplementary Material 

# Interactions between the discoidin domain receptor 1 and β1 integrin regulate attachment to collagen

## bio.20135090 Supplementary Material

**Files in this Data Supplement:**

- Supplementary Material - Lisa A. Staudinger et al. doi: 10.1242/bio.20135090
