# Supplementary material for: Interactions between the discoidin domain receptor 1 and β1 integrin regulate attachment to collagen
Source: Biol Open. 2013 Sep 13;2(11):1148–59. doi: 10.1242/bio.20135090 (PMC3828761; doi:10.1242/bio.20135090)
Supplement: Supplementary Material [file supp_bio.20135090_bio.20135090-s1.pdf]

Supplementary Material

Lisa A. Staudinger et al. doi: 10.1242/bio.20135090

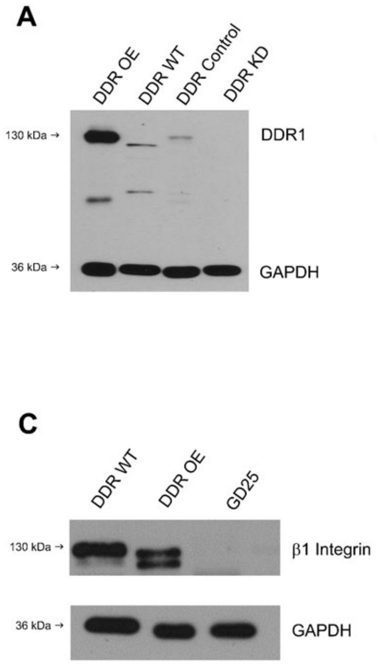

Fig. S1. (A) Whole cell lysates of mouse NIH3T3 DDR1 (b isoform) overexpressing fibroblasts (OE), or 3T3 wild-type cells, or 3T3 wild-type cells transfected with empty vector or 3T3 cells pre-incubated for 2 days with siRNA to DDR1. Cells were prepared and immunoblotted for DDR1 and co-blotted for GAPDH. (B) Immunoblots of lysates of human gingival fibroblasts treated with DDR1 siRNA (DDR KD) or with irrelevant siRNA (DDR WT) for 2 days prior to preparation. (C) Immunoblotting for total β1 integrin protein expression of whole cell lysates prepared under reducing conditions. Cells were DDR1 wild-type cells (DDRWT), DDR1 over-expressing cells (DDR OE) or GD-25 cells.

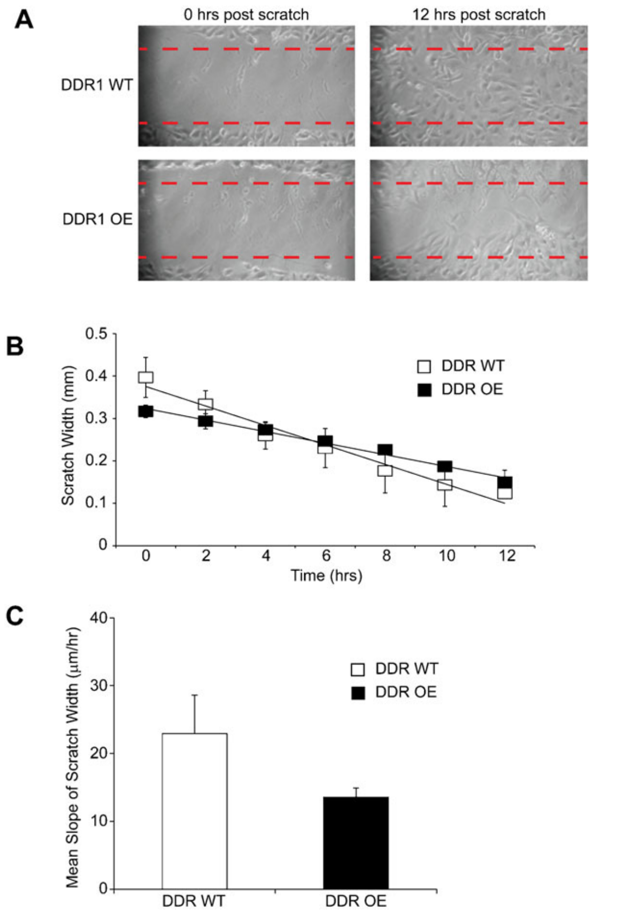

Fig. S2. DDR1 over-expression affects cell migration on collagen. (A) Representative, differential interference contrast images of wild-type or DDR1 over-expressing cells plated on collagen. Cells were examined in *in vitro* scratch assays at 0 hours and 12 hours after scratching. (B) Best linear fit of reduction of scratch width (in mm) over time. (C) Comparison of migration rates between wild-type and DDR1 over-expressing cells. Cell migration on collagen was faster in the wild-type cells compared with the DDR1 over-expressing cells ($P<0.05$). All data represent mean + S.E.M. ($n=3$ independent experiments).

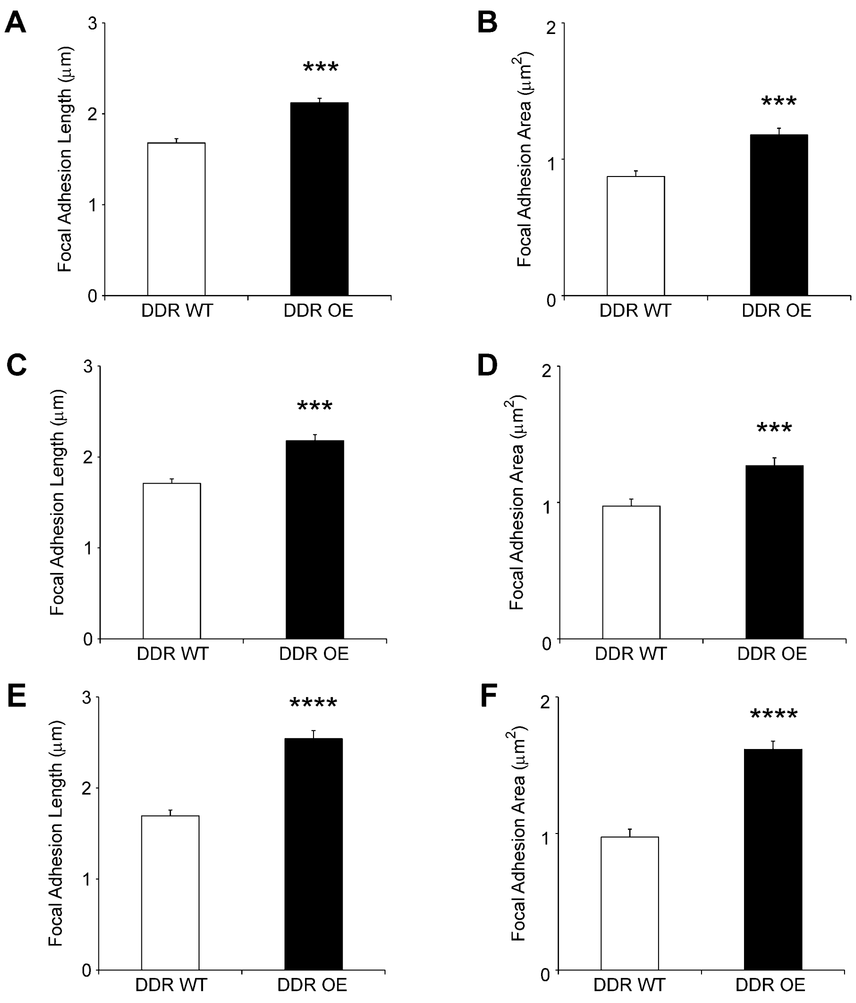

Fig. S3. DDR1 over-expression enhances focal adhesion length and area. (A) Talin-stained focal adhesions were longer ($P<0.0001$) in DDR1 over-expressing cells. (B) Talin-stained focal adhesions exhibit larger area ($P<0.0001$) in DDR1 over-expressing cells. (C) Paxillin-stained focal adhesions were longer ($P<0.0001$) in DDR1 over-expressing cells. (D) Paxillin-stained focal adhesions exhibit larger area ($P<0.0001$). In all analyses, Metamorph was used to quantify mean + S.E.M ($n=20$ cells analyzed for each cell type). (E) Vinculin-stained focal adhesions are longer ($P<0.0001$) and (F) exhibit increased area ($P<0.0001$). All analyses were done with Metamorph. Data are mean+S.E.M. ($n=20$ cells analyzed for each cell type and for each type of analysis).
